# Memory Precursors and Short-Lived Effector T cell Subsets Have Different Sensitivities to TGFβ

**DOI:** 10.3390/ijms24043930

**Published:** 2023-02-15

**Authors:** Jeremy A. O’Sullivan, Frederick J. Kohlhapp, Andrew Zloza, Lourdes Plaza-Rojas, Brianna Burke, Nickolai O. Dulin, José A. Guevara-Patiño

**Affiliations:** 1 Department of Surgery, and Cancer Biology, Loyola University Chicago, IL 60153, USA; 2 Department of Immunology, H. Lee Moffitt Cancer Center, Tampa, FL 33612, USA; 3 Department of Medicine, The University of Chicago, Chicago, IL 60637, USA

**Keywords:** TGFβ, MPEC, SLEC, CD8, Smad, RGS3, T-bet, tolerance

## Abstract

After exposure to an antigen, CD8 T cells reach a decision point about their fate: to become either short-lived effector cells (SLECs) or memory progenitor effector cells (MPECs). SLECs are specialized in providing an immediate effector function but have a shorter lifespan and lower proliferative capacity compared to MPECs. Upon encountering the cognate antigen during an infection, CD8 T cells rapidly expand and then contract to a level that is maintained for the memory phase after the peak of the response. Studies have shown that the contraction phase is mediated by TGFβ and selectively targets SLECs, while sparing MPECs. The aim of this study is to investigate how the CD8 T cell precursor stage determines TGFβ sensitivity. Our results demonstrate that MPECs and SLECs have differential responses to TGFβ, with SLECs being more sensitive to TGFβ than MPECs. This difference in sensitivity is associated with the levels of TGFβRI and RGS3, and the SLEC-related transcriptional activator T-bet binding to the TGFβRI promoter may provide a molecular basis for increased TGFβ sensitivity in SLECs.

## 1. Introduction

Soon after activation, CD8 T cells undergo a cell fate decision: to either become short-lived effector cells (SLECs, KLRG1^hi^CD127loT-bet^hi^) specialized in exerting an immediate effector function and then contract as a population or become memory progenitor effector cells (MPECs, KLRG1^lo^CD127^hi^T-bet^lo^) destined for a longer lifespan and an increased proliferative capacity. As a bulk population, CD8 T cells expand quickly upon encountering the cognate antigen during infection and then contract to levels maintained for the memory phase after the peak of the response, when the antigen has been cleared. A previous study has shown that the contraction phase is mediated by TGFβ and acts selectively on SLECs, while MPECs are spared [1]. While this effect was found to correlate with Bcl-2 levels in these populations, potential differences in the sensitivity to TGFβ signaling itself have not yet been fully explored.

TGFβ has been shown to play a role in cellular differentiation, wound healing, and immune regulation [2,3,4,5,6]. TGFβ signals through the TGFβRI and TGFβRII complex, which phosphorylates the receptor-regulated Smad signaling proteins (R-Smads), Smad2, and Smad3. Smad2 or Smad3 then complexes with the co-Smad, Smad4, and, together, this complex translocates to the nucleus to activate TGFβ-responsive gene transcription [7,8]. The activation of TGFβ-induced gene transcription is prevented by the regulator of G protein signaling-3 (RGS3) by binding to Smad2, Smad3, or Smad4 [9]. Given the importance of SLECs and MPECs, the aim of this study is to investigate how the CD8 T cell precursor stage determines TGFβ sensitivity.

Here, we report on MPECs and SLECs’ differential responses to TGFβ. In contrast to MPECs, SLECs effectively respond to TGFβ. The levels of TGFβRI and RGS3 were associated with this difference in sensitivity to TGFβ. Furthermore, the SLEC-related transcriptional activator T-bet binds to the TGFβRI promoter, providing a potential molecular basis for augmented TGFβRI levels and increased TGFβ sensitivity in SLECs.

## 2. Materials and Methods

### 2.1. Mice

Six-week-old, specific-pathogen-free C57BL/6 (B6) and C57BL/6-Tg (TcraTcrb)1100Mjb/J (OT-I) mice were purchased from Jackson Laboratories. B6 mice were used to generate wild-type open repertoire T cells. OT-I mice were used as a source of CD8 T cells with a given TCR specificity. Alternative in vitro models were incorporated into the study, when possible. Animal studies were performed in accordance with Loyola’s and Moffitt’s Institutional Animal Care and Use Committee (IACUC), #205488, 212646.

### 2.2. In Vitro Activation

All cells were cultured in RPMI supplemented with 10% heat-inactivated Fetal Bovine Serum (Atlanta Biologicals, Lawrenceville, GA, USA), 2mM L-glutamine (Mediatech, Manassas, VA, USA), and 1% penicillin/streptomycin (Mediatech, Manassas, VA, USA). CD8 T cells were activated with 100 ng/mL OVA_257_ peptide (SIINFEKL, New England Peptide, Gardner, MA, USA), 1 µg/mL anti-CD3ε (clone 145-2C11, Bio X Cell, West Lebanon, NH, USA), or 1 µg/mL anti-CD3ε and 5 µg/mL anti-CD28 (clone 37.51, Bio X Cell) for 5 days in media supplemented with 30 U/mL IL-2 (R&D Systems, Minneapolis, MN, USA). When noted, cells were incubated with TGFβ1 (EMD Chemicals, Inc., Gibbstown, NJ, USA) at a final concentration of 20 ng/mL.

### 2.3. Antibodies and Flow Cytometry

Flow cytometry antibodies for anti-CD127 (A7R34) and CD122 (2-1221-82) were purchased from eBioscience (San Diego, CA, USA): anti-CD3ε (BD Biosciences, San Diego, CA, USA), anti-CD8 (4B11) (Invitrogen, Carlsbad, CA, USA), anti-KLRG1 (2F1) (Southern Biotech, Birmingham, AL, USA), anti-TGFβRI, anti-Smad2/3, anti-Smad4 (Santa Cruz Biotechnology, Santa Cruz, CA, USA), anti-TGFβRII (R&D Systems), anti-T-bet (BioLegend, San Diego, CA, USA), and anti-RGS3. The anti-RGS3 antibody has been described previously [10]. DAPI was purchased from Invitrogen. Extracellular and intracellular marker staining was performed as previously described [11]. Flow cytometric analysis was performed using an LSRII flow cytometer (Becton Dickinson, Franklin Lakes, NJ, USA). Data were analyzed using Flow Jo software (Tree Star, Ashland, OR, USA). The cells were fixed and permeabilized with methanol and formaldehyde prior to staining for analysis with the Amnis Imagestream imaging flow cytometer. Data were analyzed using Amnis IDEAS software v4.0 (Amnis Corporation, Seattle, WA, USA).

### 2.4. Bioinformatics

Sequences for the TGFβRI and IFN-γ promoters were found, and T-bet binding sites and modules were identified using the Genomatix software suite (Genomatix Genome Analyzer, GmbH, Munich, Germany). The retrieval and analysis of promoter regions were conducted using the Gene2Promoter Mus musculus genome (Genomatix Genome Analyzer, GmbH, Munich, Germany).

### 2.5. Chromatin IP

Polyclonal CD8 T cells were activated with anti-CD3ε and anti-CD28 mAbs, purified by positive selection with MACS CD8a microbeads (Miltenyi Biotec, Auburn, CA, USA), and lysed. ChIP was performed with Millipore (Billerica, MA, USA) EZ-ChIP reagents, according to the manufacturer’s specifications, and with the anti-T-bet mAb from Santa Cruz Biotech. The presence of the TGFβRI promoter fragment was confirmed by PCR with the following primers: forward 5’-ACA CTT TGG GCT CGA ACT TG-3’ and reverse 5’-AGA CCA GTG CCA AAT GGA AG-3’.

## 3. Results and Discussion

### 3.1. SLECs Exhibit a More TGFβ-Sensitive Phenotype

**Figure 1:**
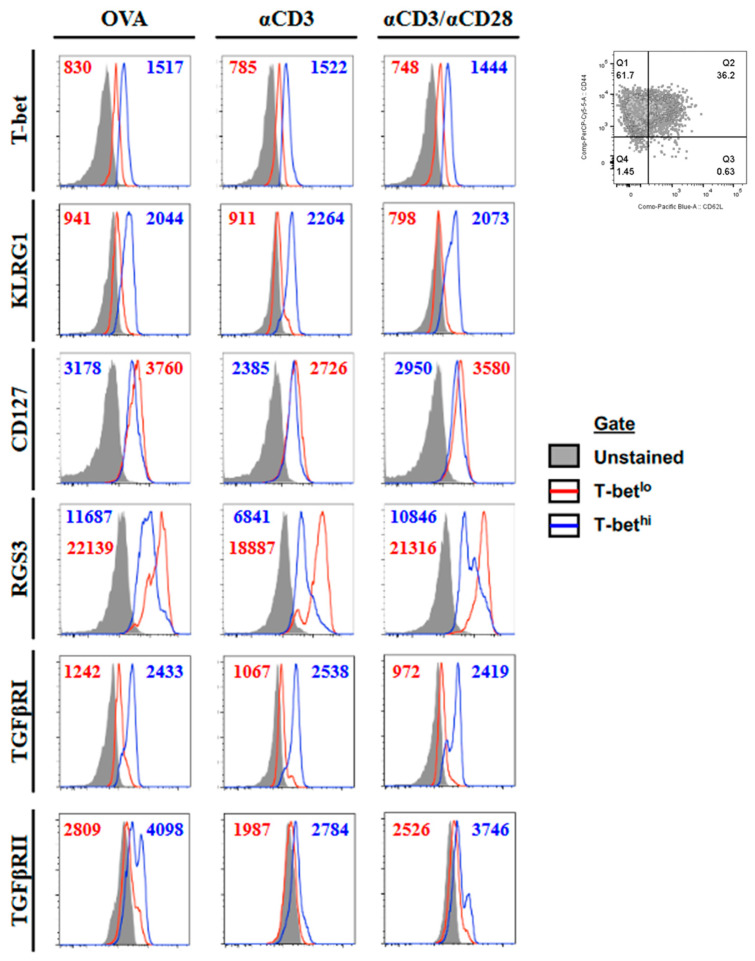
Phenotype of T-bet^hi^ and T-bet^lo^ effector CD8 T cells. OT-I splenocytes were activated in vitro for 5 days with either OVA_257_, anti-CD3ε mAb, or anti-CD3ε and anti-CD28 mAbs. Cells were gated on CD8 CD44^hi^ (upper right) and T-bet^hi^ or T-bet^lo^ (gated from T-bet vs. CD8 plot). Histograms demonstrate the expression levels of T-bet, KLRG1, CD127, TGFβRI, TGFβRII, and RGS3 in unstained controls (gray-filled histograms) and T-bet^lo^ (red line) and T-bet^hi^ (blue line) CD8 effector T cells. Mean fluorescence intensities are shown for T-bet^lo^ (red text) and T-bet^hi^ (blue text) CD8 T cells for each plot. The data shown are representative of at least three individual experiments with similar results.

**Figure 2:**
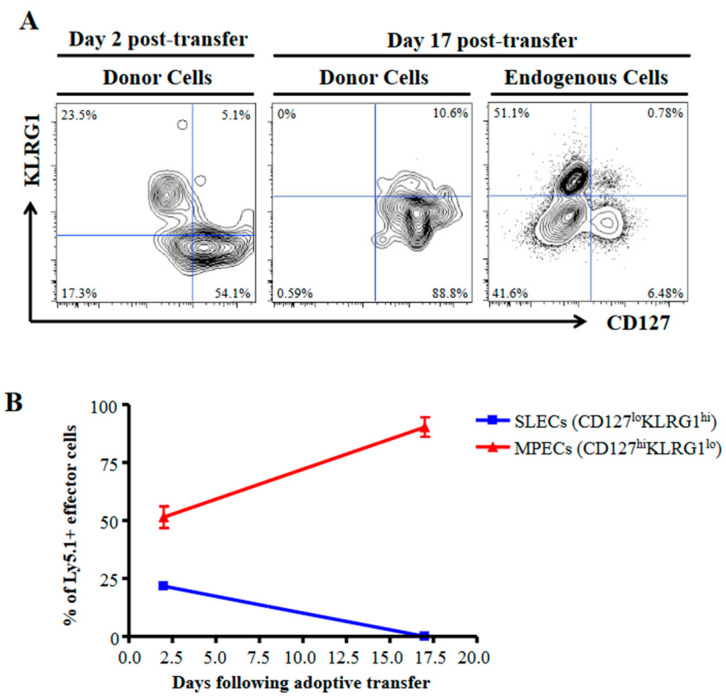
MPECs and SLECs generated in vitro possess characteristic persistence properties in vivo. (**A**,**B**) Congenically marked (Ly5.1+) polyclonal CD8 T cells were stimulated with anti-CD3ε and anti-CD28 mAbs for 5 days prior to transfer into B6 (Ly5.2+) mice. The persistence of donor MPECs and SLECs was followed by a blood draw (at days 2 and 17) and splenocyte harvest (at day 17) until SLECs were no longer detectable. The donor cells constitute 3–5% of the total in the recipient mice. Representative flow plots (**A**) and the mean percentage of MPECs (red line) and SLECs (blue line) across time (**B**) are shown. Both donor and endogenous cells are gated on effector CD8 T cells. Error bars represent the standard deviation.

To investigate the basis for the differential sensitivity to TGFβ between MPECs and SLECs populations, we used the transcription factor T-bet as a marker. T-bet has been shown to be upregulated in SLECs relative to MPECs and appears to underlie much of the SLEC phenotype [12,13]. We generated effector cells by activating OT-I CD8 T cells in vitro for 5 days with the OVA257 peptide, anti-CD3ε, or anti-CD3ε and anti-CD28. Each resulting effector population (CD44hi) contained cells that were T-bethi and T-betlo (Figure 1). Furthermore, those identified as T-bethi had upregulated KLRG1 and slightly downregulated CD127, identifying them as SLECs (Figure 1). Next, we compared their ability to survive in time after in vivo transfer. These SLECs, as expected, failed to persist in vivo following the adoptive transfer. In comparison, those expressing the MPEC phenotype were preferentially maintained (Figure 2). To ascertain whether the differences in TGFβ signaling between MPECs and SLECs may underlie their different responses to TGFβ during the contraction phase, we analyzed the expression levels of TGFβRI, TGFβRII, and RGS3, an inhibitor of TGFβ signaling [9]. There is evidence for the modulation of TGFβ receptors, e.g. TGFβRI in intestinal epithelial cells by polyamine depletion [14], and of RGS molecules in dendritic cells (DCs) and B cells in response to activation signals [15,16]. While we observed no significant differences in TGFβRII, we found that TGFβRI was downregulated and that RGS3 was upregulated in MPECs (Figure 1). These results strongly suggest that different sensitivities to TGFβ signaling may underlie different responses to TGFβ between MPECs and SLECs.

### 3.2. TGFβ-Induced Smad Nuclear Translocation Is Decreased in MPECs Relative to SLECs

**Figure 3:**
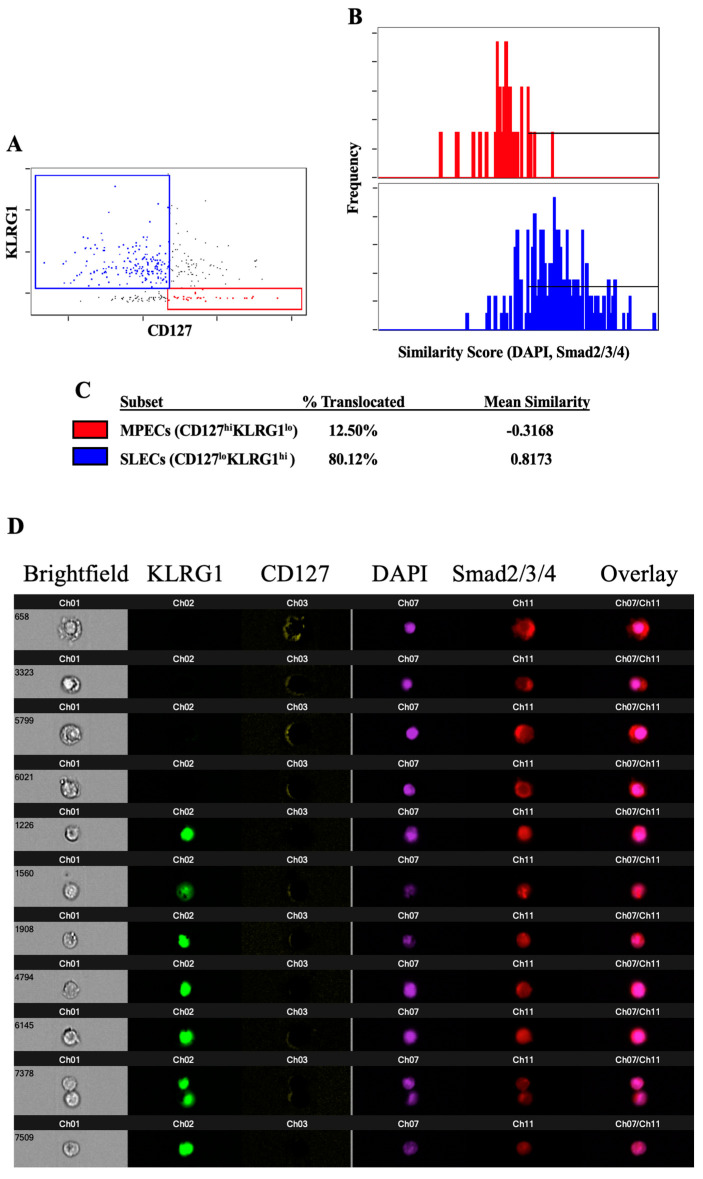
Smad proteins do not efficiently translocate to the nucleus in response to TGFβ signaling in MPECs. Polyclonal CD8 T cells were stimulated with anti-CD3ε and anti-CD28 mAbs for 5 days prior to a 4 h incubation with TGFβ. The cells were then fixed, stained, and analyzed for the nuclear translocation of the Smad2/3/4. (**A**) Plot of KLRG1 and CD127 demonstrating the gating of MPECs (red) and SLECs (blue). (**B**) Plot of similarity values between fluorescence indicating the location of Smad2/3/4 and fluorescence indicating the location of the nucleus (DAPI) in MPECs (red) and SLECs (blue). Horizontal line indicates the similarity values that are consistent with the Smad translocation to the nucleus. (**C**) Calculated percentage of MPECs and SLECs in which Smad2/3/4 have translocated to the nucleus, according to the gate shown in (**B**), and the mean similarity value for each population. (**D**) Representative images showing the intensity and location of KLRG1, CD127, the nucleus, Smad2/3/4, and an overlay of the nuclear and Smad images. The data shown are representative of at least three individual experiments with similar results.

To measure the TGFβ sensitivity in MPECs and SLECs, we sought to determine whether downstream TGFβ signaling occurred in both populations to the same degree. For TGFβ to exert its transcriptional regulatory function, R-Smads (Smad2 and Smad3) must form a complex with Smad4 and then enter the nucleus. To examine if TGFβRI and RGS3 expression levels influence the ability of Smad signaling proteins to translocate to the nucleus, we analyzed the nuclear localization of the Smad proteins in both MPECs and SLECs. This analysis was important, as variations in TGFβRI and RGS3 levels would be expected to affect the nuclear translocation of the Smad signaling proteins. CD8 T cells were activated for 5 days with anti-CD3ε and anti-CD28 mAbs, incubated with TGFβ, and stained for CD8, the MPEC/SLEC surface markers CD127 and KLRG1, Smad2/3/4, and the nucleus. The cells were analyzed with an imaging flow cytometer to be able to discern not only the fluorescence intensity of the surface markers but also the location of the Smad signaling proteins with respect to the nucleus. We observed more of a translocation of Smad2/3/4 to the nucleus downstream of TGFβ signaling in cells expressing an SLEC phenotype (KLRG1^hi^CD127^lo^) than in cells expressing an MPEC phenotype (KLRG1loCD127^hi^) (Figure 3A–D). We conclude that MPECs are less sensitive to TGFβ signaling than SLECs. An analysis of the expression levels of Smad7, itself a target of TGFβ-induced, Smad-mediated transcription, revealed no significant differences between MPECs and SLECs (data not shown). However, the influence of other factors on Smad7 expression at the transcriptional or post-transcriptional levels, e.g., the ubiquitination of Smad7 by Smurf1/2, Jab1/CSN5, or Arkadia, may cause Smad7 to be an inaccurate indicator of the level of TGFβ signaling in these cells [17,18,19].

### 3.3. T-Bet Binds to the TGFβRI Promoter

**Figure 4:**
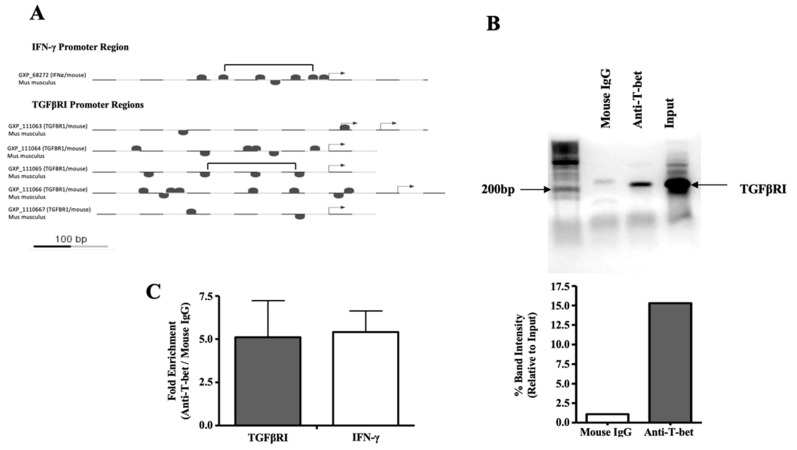
T-bet binds to the TGFβRI promoter. (**A**) Putative T-bet binding sites in the TGFβRI promoter region are marked. For comparison, similar sites in the IFN-γ promoter region are also indicated. Regions containing similar T-bet binding modules are denoted in brackets. Arrows denote transcription start sites. (**B**,**C**) Polyclonal CD8 T cells were stimulated with anti-CD3ε and anti-CD28 mAbs for 5 days prior to lysis and analysis. Chromatin was immunoprecipitated using an anti-T-bet mAb and a control mouse IgG mAb. Primers specific for the TGFβRI promoter region containing the module with similarity to the IFN-γ promoter were used to amplify a 214 bp fragment. (**B**) Band intensities relative to the input are shown. (**C**) Primers specific to regions of the TGFβRI and IFN-γ promoters were used to measure abundance by real-time PCR following control mouse IgG or anti-T-bet chromatin immunoprecipitation. The mean fold enrichment for each promoter is noted. Error bars represent the standard deviation.

As T-bet determines much of the SLEC phenotype, we sought to determine if it could also be transcriptionally involved in generating the phenotype of enhanced sensitivity to TGFβ. There have been indications that T-bet may be involved in sensitizing T cells to TGFβ-mediated regulation. In a study evaluating the susceptibility of CD4 T cells to TGFβ-mediated regulation, memory Th1 (T-bet+) cells were found to be susceptible to regulation, whereas memory Th2 (T-bet-) cells were found to be resistant [20,21]. To answer this question, we searched for putative T-bet binding sites in the TGFβRI promoter. We found a T-bet binding module similar to one found in the IFN-γ promoter, in which cooperative T-bet binding sites occur at regular intervals [22] (Figure 4A). To test whether the putative binding sites near or within this module were actual T-bet binding sites, we polyclonally activated CD8 T cells as before and immunoprecipitated chromatin with an anti-T-bet mAb. With primers specific to the region of the TGFβRI promoter with similarity to the IFN-γ promoter, we confirmed that T-bet does in fact bind to the TGFβRI promoter (Figure 4B).

**Figure 5:**
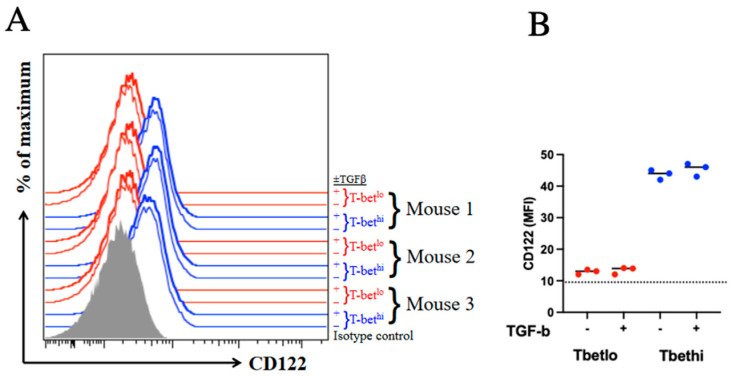
CD122 expression is not significantly affected by TGFβ signaling. Endogenous MPECs and SLECs were incubated with or without 20 ng/mL TGFβ for 2 h prior to the analysis of CD122 expression by flow cytometry (**A**). Gray-filled histogram represents isotype control. T-bet^hi^ SLECs and T-bet^lo^ MPECs from individual mice are represented by blue and red histograms, respectively. (**B**) CD122 MFI are compared between groups; *p* > 0.05. The experiment was conducted using three mice per group and repeated twice.

T-bet has previously been shown to modulate the expression of CD122 and thus enhance the sensitivity to IL-15 signaling in CD8 T cells [13,23]. Furthermore, TGFβ has been shown to downregulate T-bet in Th1 cells [24,25]. To determine if TGFβ exerts its apoptotic effect on SLECs through the downregulation of T-bet and, thus, CD122, we analyzed CD122 expression in both SLECs and MPECs in the presence and absence of TGFβ. Although CD122 was indeed upregulated in SLECs when compared to MPECs, as expected, neither population downregulated CD122 in response to TGFβ signaling, which is consistent with the lack of modulation of T-bet levels by TGFβ (Figure 5).

## 4. Discussion

Studies of autoimmunity, transplantation, and tumor immunity, in which TGFβ plays a prominent role in tolerance and immune suppression, suggest that MPECs and the resulting memory cells are less sensitive to TGFβ signaling than their SLEC counterparts. Gattinoni et al. have shown that early effector CD8 T cells (similar to MPECs, with higher levels of CD127) are better able to reject a large, established B16 murine melanoma tumor than intermediate or full effector CD8 T cells (similar to SLECs, with higher levels of KLRG1) [26]. Furthermore, Yang et al. have shown that memory T cells are resistant to regulatory T cell (Treg)-mediated tolerance induction in a skin graft model in which naive T cells are sensitive [27]. TGFβ signaling is critical to both the melanoma B16- and Treg-mediated suppression of T cell responses. Furthermore, cells treated with TGFβ before the transfer in the adopted therapy of cancer appear to be more effective, as one might expect, if the TGFβ treatment selectively eliminated SLECs and thus enriched the population for cells not able to respond as effectively to TGFβ [28]. In addition, a transcriptional signature associated with central memory in autoimmune CD8 T cells has been shown to correlate with a poorer prognosis in two autoimmune diseases: antineutrophil cytoplasmic antibody (ANCA)-associated vasculitis (AAV) and systemic lupus erythematosus (SLE) [29]. Our results suggest a novel mechanism whereby SLECs are more sensitive and MPECs are less sensitive to TGFβ. Together, these results demonstrate that MPECs are less sensitive to TGFβ than SLECs, which provides an alternative explanation for how MPECs escape cell death during the contraction phase. Additionally, our results suggest that the transcription factor T-bet may promote TGFβ sensitivity in SLECs. This contrasts with the hypothesis that the Bcl-2 levels of MPECs and SLECs determine the susceptibility to TGFβ-mediated contraction, which we believe is made less feasible by studies that indicate that the constitutive expression of CD127 (IL7Rα) and Bcl-2 overexpression fail to save SLECs during the contraction phase [30,31].

These results have important implications in cancer immunotherapy and the treatment of autoimmune diseases, as TGFβ-insensitive cells would be ideal candidates for the adopted therapy of cancer and priority targets for the therapy of autoimmunity and graft immunity.

## Data Availability

Data will be available to investigators upon request.

## Abbreviations

DAPI	4’,6-diamidino-2-phenylindole
KLRG1	killer cell lectin-like receptor subfamily G member 1
mAb	monoclonal antibody
MPEC	memory progenitor effector cell
RGS3	regulator of G protein signaling 3
SLEC	short-lived effector cell
T-bet	T-box expressed in T cells
TGFβ	transforming growth factor β
TGFβRI	TGFβ receptor I
TGFβRII	TGFβ receptor II
Th1/Th2	T helper c regulatory T cell ell type 1/2
Treg	regulatory T cell
